# Integrated RNA-Seq and sRNA-Seq Analysis Identifies Chilling and Freezing Responsive Key Molecular Players and Pathways in Tea Plant (*Camellia sinensis*)

**DOI:** 10.1371/journal.pone.0125031

**Published:** 2015-04-22

**Authors:** Chao Zheng, Lei Zhao, Yu Wang, Jiazhi Shen, Yinfei Zhang, Sisi Jia, Yusheng Li, Zhaotang Ding

**Affiliations:** 1 Tea Research Institute, Qingdao Agricultural University, Qingdao, Shandong, China; 2 Key Laboratory of Genetic Improvement and Breeding for Horticultural Plants, Qingdao, Shandong, China; 3 Fruit and Tea Technology Extension Station, Jinan, Shandong, China; Institute of Hydrobiology, Chinese Academy of Sciences, CHINA

## Abstract

Tea [*Camellia sinensis* (L) O. Kuntze, *Theaceae*] is one of the most popular non-alcoholic beverages worldwide. Cold stress is one of the most severe abiotic stresses that limit tea plants’ growth, survival and geographical distribution. However, the genetic regulatory network and signaling pathways involved in cold stress responses in tea plants remain unearthed. Using RNA-Seq, DGE and sRNA-Seq technologies, we performed an integrative analysis of miRNA and mRNA expression profiling and their regulatory network of tea plants under chilling (4℃) and freezing (-5℃) stress. Differentially expressed (DE) miRNA and mRNA profiles were obtained based on fold change analysis, miRNAs and target mRNAs were found to show both coherent and incoherent relationships in the regulatory network. Furthermore, we compared several key pathways (e.g., ‘Photosynthesis’), GO terms (e.g., ‘response to karrikin’) and transcriptional factors (TFs, e.g., DREB1b/CBF1) which were identified as involved in the early chilling and/or freezing response of tea plants. Intriguingly, we found that karrikins, a new group of plant growth regulators, and β-primeverosidase (BPR), a key enzyme functionally relevant with the formation of tea aroma might play an important role in both early chilling and freezing response of tea plants. Quantitative reverse transcriptase-polymerase chain reaction (qRT-PCR) analysis further confirmed the results from RNA-Seq and sRNA-Seq analysis. This is the first study to simultaneously profile the expression patterns of both miRNAs and mRNAs on a genome-wide scale to elucidate the molecular mechanisms of early responses of tea plants to cold stress. In addition to gaining a deeper insight into the cold resistant characteristics of tea plants, we provide a good case study to analyse mRNA/miRNA expression and profiling of non-model plant species using next-generation sequencing technology.

## Introduction

Tea (*Camellia sinensis* (L.) O. Kuntze) is the most popular health drink in the world, and the tea plant is one of the most important commercial beverage crops in Kenya, China, Sri Lanka, India, among others [1]. The tea plant, as a perennial evergreen woody crop, is constantly exposed to environmental changes, requiring transient, rapid and seasonal responses [2]. Every year significant loss of tea production can result from sudden frosts in fall, unusual freezing stress in winter and early spring frosts. Furthermore, to ensure a prolonged growth period in the high-latitude regions, novel tea plant cultivars with improved cold resistance are in demand.

Cold stress, including chilling (0–15°C) and freezing (< 0°C) temperatures, is a serious environmental stress that disrupts cellular homeostasis and limits plant growth. During the cold stress process, a series of protective mechanisms are triggered while a series of negative effects occur, including the reduced nutrient absorption rates, reduced net and maximal photosynthetic rates, and the inhibition of plant growth [3]. On the other hand, the physiological and biochemical status of cold-stressed plants are also altered, such as transient increases in hormone levels [4] and alternating the composition of the membrane lipid [5]. Furthermore, the accumulation of numerous cryoprotectants, such as glycine betaine, proline and trehalose [6], and the enhancement of antioxidative mechanisms [7] are also occurred. These changes are, at least in part, mediated through the differential expression of many genes [8, 9]. These genes are thought to be induced either by cold *per se* or by the relative state of dehydration that is brought about by cold stress [10]. Many of these COR (cold-regulated) genes have been identified by transcriptome analysis. In *Arabidopsis*, for example, several hundred transcripts have been reported to respond to cold [11, 12]. Similarly, in the woody plants, a large number of genes have also been shown to be cold responsive (e.g., *A*. *mongolicus* [13] and *J*. *curcas* [14]). To date, some COR genes have been isolated from tea plants [15, 16]. Recently, Wang *et al*. (2013) have presented the first whole transcriptome analysis of tea plants during the cold acclimation process using RNA-Seq combined with digital gene expression (DGE) analysis [17]. A large number of cold-responsive genes involved in diverse biological processes or pathways were identified during the cold acclimation process. However, the whole transcriptome-level changes of early cold response in tea plants remain unclear, and the differences between chilling and freezing response mechanisms in tea plants are far from being fully understood. Moreover, cold perception is also associated with changes at the post-translational levels [18]. Numerous cold-responsive transcription factors (TFs), such as AP2-EREBP (APETALA2/ET-Responsive Element Binding Protein), MYB (Myeloblastosis), NAC (NAM, ATAF1/2, CUC2), WRKY (named after the WRKY amino acid motif), and bHLH (basic Helix-Loop-Helix) have been studied extensively in model plants [19, 20]. In addition to transcription factors, other signaling components mediating cold response process, including kinases, enzymes involved in hormone biosynthesis and Ca^2+^ sensors, are also regulated at the post-translational level [21].

MicroRNAs (miRNAs) are endogenous single-stranded non-coding small RNAs (sRNAs) of approximately 21 nt in length; these RNAs regulate gene expression at the post-transcriptional level through base-pairing to target mRNAs [22, 23]. To date, increasing number of studies indicate that plant miRNAs are involved in various biotic and/or abiotic stress responses such as drought [24, 25], high salinity [25], extreme temperatures [26, 27], nutrient deficiency [28], and pathogens [29]. Though numbers of miRNAs were identified from various crop plant species, there was still no registered *C*. *sinensis* miRNA in miRBase (Release 20.0, http://www.mirbase.org/) [30]. Recently, Mohanpuria and Yadav [31] discovered six novel small RNAs candidates from tea using a direct cloning approach for the first time. Das and Mondal [32], Prabu and Mandal [33] and Zhu and Luo [34] identified several miRNAs from tea plants by expressed sequence tag (EST) analysis, and more attention was given to the function of tea miRNAs. Most recently, Anburaj *et al*. analysed the function of several tea miRNAs within the bud tissues using stem-loop pulse RT-qPCR method [35]. Despite these efforts, our knowledge of the role played by miRNAs in tea plants’ cold stress response is still limited at the whole-genome level. Moreover, little attention has been paid to understanding how genes, transcription factors and miRNAs expression are integrated into the dynamic and complex regulatory network which act together to alter regulation and finally contribute to the enhancement of chilling and freezing tolerance in tea plants.

Insights in miRNA-mRNA regulatory networks facilitate the understanding of fine-tuning of gene expression at post-transcriptional level. However, in silico predictions of miRNA-mRNA interactions do not take into account the specific transcriptomic status of the biological system and are biased by false positives. For example, miRNAs may indirectly interact with mRNAs through other TFs, miRNAs or endo-siRNAs (e.g. trans-acting siRNAs, ta-siRNAs) besides direct base pairing [36, 37]. Consequently, indirect mRNA modulatory effects of miRNAs to decrease or increase target mRNAs may be frequent or even greatly outnumber direct target suppressions [38]. One possible solution to predict rather reliable miRNA-mRNA relations in the specific biological context is to integrate real mRNA and miRNA transcriptomic data as well as in silico target predictions. Next-generation sequencing technologies such as RNA-Seq and DGE have proved to be cost-effective and high-throughput means to analyze the transcriptome in non-model species [17, 39, 40]. RNA-Seq approach can also be served to analyze the sRNAs of the transcriptome when libraries are constructed from low-molecular weight RNA fractions [41]. These technologies offer several advantages compared with existing technologies such as EST sequencing and microarrays [39, 42]. Here, we provided a two-dimensional map of the mRNA and miRNA expression profiles on tea leaves subjected to chilling (4°C) and freezing (-5°C) temperatures for 4- or 8-h. We identify a complex and dynamic network of genes, transcription factors and miRNAs involved in the regulation of early cold response. In addition to gaining a deeper insight into the molecular mechanisms of early cold response, the differences between chilling and freezing response in tea plants are discovered. The results of this study should contribute to biotechnological manipulation with the aim of improving the cold resistance of tea plants.

## Results

### Analysis of transcriptome sequencing

#### Sequencing and assembly

RNA isolated from the control (25°C, CK) and four cold-treated [chilling treatment (4°C, CT) for 4- and 8-h (CT4 and CT8); freezing treatment (-5°C, FT) for 4- and 8-h (FT4 and FT8)] tea leaves were equally mixed to construct a broad cDNA library associated with cold response. Overviews of the sequencing and assembly results were shown in Table 1 and Table 2, respectively. After discarding the low-quality raw reads, 138,748,418 clean reads were remained. Through the Trinity *de novo* assembly method, we obtained 218,421 transcripts, and 102,587 non-redundant unigenes were achieved with a N50 of 1,009 bp and an average length of 651 bp (S1 Fig).

**Table 1:** Quality of sequencing.

Sample	Raw Reads	Clean reads	Clean bases	Error (%)	Q20 (%)	Q30 (%)	GC (%)
**Cs_R1**	71,247,895	69,374,209	6.94G	0.03	97.78	92.87	44.02
**Cs_R2**	71,247,895	69,374,209	6.94G	0.03	97.41	92.35	44.03

R1: Reads sequencing from the left; R2: Reads sequencing from the right.

**Table 2:** Length distribution of assembled transcripts and unigenes.

Nucleotides length (bp)	Transcripts	Unigenes
**200–500 bp**	94,078	67,432
**500–1 kbp**	44,230	18,788
**1k-2 kbp**	44,819	10,083
**>2 kbp**	35,294	6,284
**Total**	218,421	102,587
**Minimal length (bp)**	201	201
**Maximal length (bp)**	16,661	16,661
**N50 (bp)**	1,866	1,009
**Average length (bp)**	1,073	651

#### Functional annotation and classification

All the 102,587 assembled unigenes were searched against Nr, Nt, Swiss-Prot, KOG, Pfam and KEGG databases. A total of 35,832 (34.92%) unigenes had at least one hit in BLAST search (Table 3). Among them 30,241 unigenes (29.47%) showed high homology with sequences in the Nr database, and 20,089 unigenes (19.58%) had BLAST hit in the Swiss-Prot database. The number of unigenes with significant similarity to sequences in Nt, KOG, Pfam and KEGG databases was 15,037 (14.65%), 9,841 (9.59%), 22,182 (21.62%) and 7,849 (7.65%), respectively (Table 3).

**Table 3:** Summary for the annotation of unigenes.

	Sequences (n)	Frequency(%)
**All assembled unigenes**	102,587	100
**Annotated in Nr**	30,241	29.47
**Annotated in Nt**	15,037	14.65
**Annotated in Swiss-Prot**	20,089	19.58
**Annotated in GO**	25,178	24.54
**Annotated in KOG**	9,841	9.59
**Annotated in KEGG**	7,849	7.65
**Annotated in Pfam**	22,182	21.62
**All annotated unigenes**	35,832	34.92

As per the Gene ontology (GO) classification, the 25,178 matched unigenes were classified into 3 functional categories: molecular function, biological process and cellular component (Table 3 and S2A Fig). Genes involved in ‘cellular process’ (23.21%) and ‘metabolic processes’ (21.46%) groups were notably represented in the biological process category. Under the molecular function category, ‘binding’ (46.47%) and ‘catalytic activity’ (38.35%) were the highly represented groups. In the category of cellular component, unigenes belonged to ‘cell’ (19.71%) and ‘cell part’ (19.66%) groups were highly represented (S2A Fig).

The 9,841 matched unique sequences were clustered into 26 categories, using euKaryotic Ortholog Groups (KOG) proteins (Table 3, S2B Fig). The largest group was the category for ‘general function prediction only’ (21.03%); followed by ‘post-translational modification, protein turnover and chaperon’ (12.93%); the third category was ‘signal transduction’ (8.39%) (S2B Fig).

Similarly, Kyoto Encyclopedia of Genes and Genomes (KEGG) classification was found for 7,849 unigenes that were further classified into 5 biochemical pathways, including environmental information processing (662), genetic information processing (1,794), cellular processes (860), organism system (1,289), and metabolism (3,701) (Table 3, S2C Fig). The largest group, the metabolic pathway, was well represented among the 3,701 unigenes of *C*. *sinensis*, and ‘carbohydrate metabolisms’ (20.99%), ‘energy metabolism’ (16.91%), and ‘amino acid metabolisms’ (14.83%) occupied the top three categories of metabolism pathway. Dominant categories of cellular processes, organism system, genetic information processing and environmental information processing biochemical pathways were ‘transport and catabolism’ (48.37%), ‘environmental adaptation’ (21.88%), ‘translation’ (41.19%), and ‘signal transduction’ (89.88%), respectively (S2C Fig).

### Digital gene expression (DGE) analysis among five samples

To explore the molecular mechanisms of tea plants respond to chilling and freezing stress, DGE analysis was performed to determine the differentially expressed (DE) mRNAs under chilling and freezing temperature. Approximately 13.6, 11.2, 12.8, 11.9 and 11.8 million clean reads were obtained in CK, CT4, FT4, CT8 and FT8 samples, respectively (Table 4). Gene annotation was carried out by read mapping to the 102,587 non-redundant unigenes from aforementioned RNA-Seq-based transcriptome. Accordingly, the results showed that 93.20, 93.30, 92.95, 93.38 and 93.03% of all distinct clean reads within different group could be individually mapped to the reference transcriptome, respectively (Table 4).

**Table 4:** Summary for DGE datasets.

Sample	Raw Reads	Clean reads	Clean bases	Error (%)	Q20 (%)	Q30 (%)	GC (%)	Total mapped
**CK**	13,639,661	13,624,614	0.68G	0.01	98.60	95.69	44.02	12,697,976 (93.20%)
**CT4**	11,171,035	11,160,616	0.56G	0.01	98.77	96.04	43.70	10,413,836 (93.30%)
**CT8**	12,844,582	12,826,516	0.65G	0.01	98.43	95.51	43.61	11,923,013 (92.95%)
**FT4**	11,948,725	11,937,210	0.60G	0.01	98.62	95.79	43.80	11,145,898 (93.38%)
**FT8**	11,809,267	11,795,258	0.59G	0.01	98.45	95.52	43.52	10,973,611 (93.03%)

**Fig 1:**
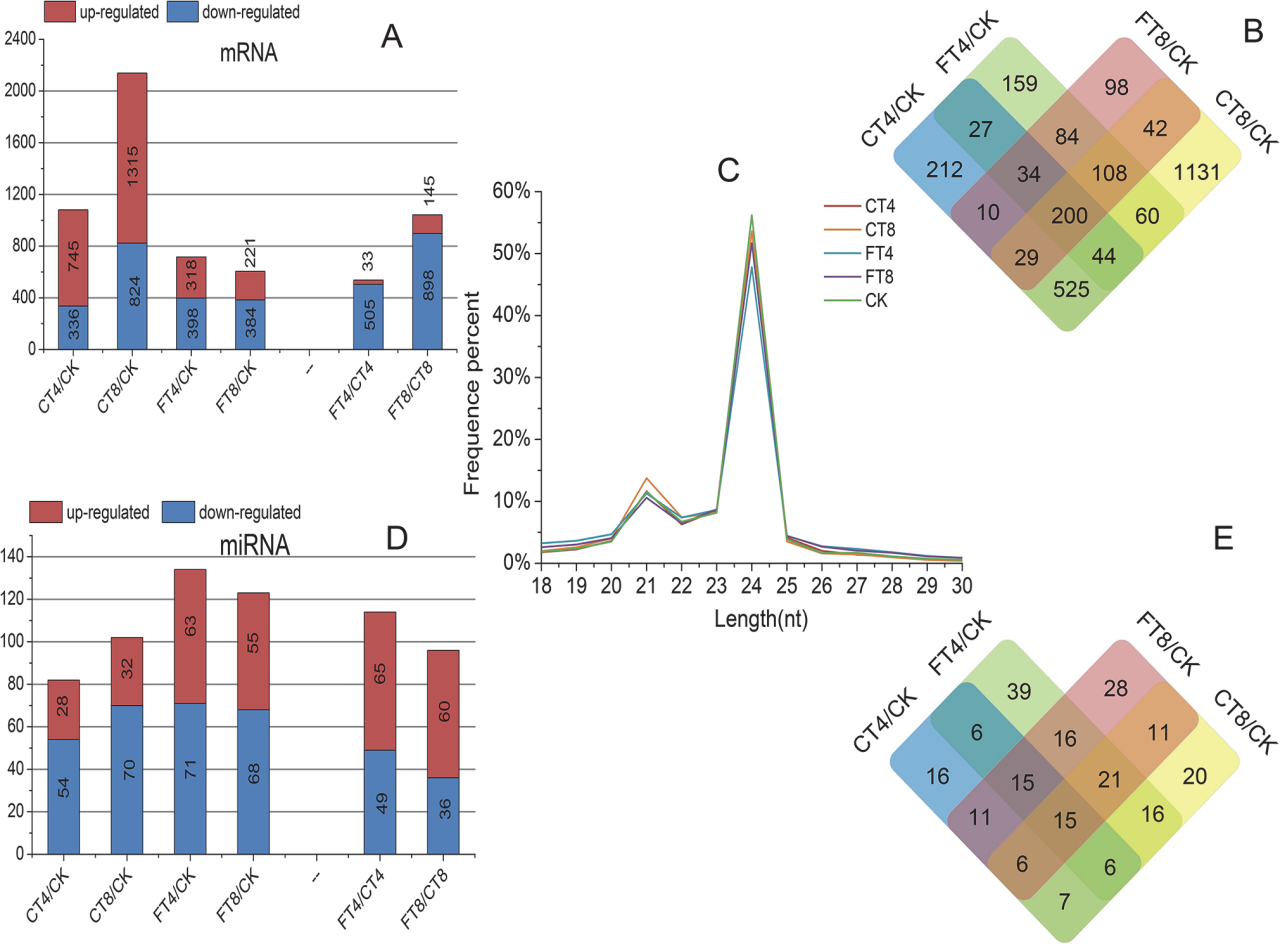
Overview of DE mRNAs, DE miRNAs and length distribution of the small RNA among libraries. The histograms showing the number of DE mRNAs (A) and miRNAs (D) among libraries. The Venn diagrams showing the overlaps among four comparisons of DE mRNAs (B) and miRNAs (E). (C) refers to the length distribution of the small RNA in different libraries.

**Fig 2:**
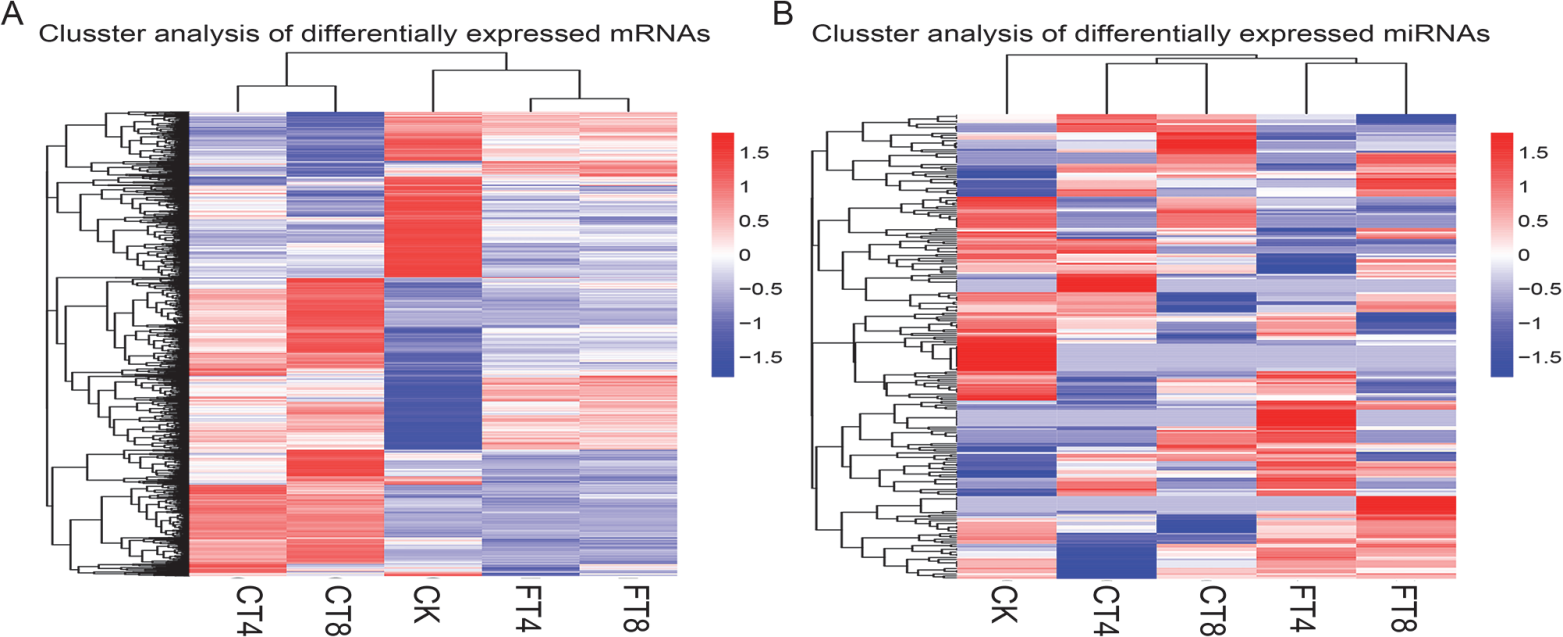
Hierarchical clustering of DE mRNAs and DE miRNAs among libraries. Hierarchical clustering of the DE mRNAs (A) and DE miRNAs (B), using the DGE and sRNA-Seq data derived from five samples (CT4, CT8, FT4, FT8 and CK samples) based on log_10_ (RPKM+1) and log_10_ (TPM+1) values, respectively.

Screening of DE mRNAs, the results showed that the gene expression level changed over time and temperature of cold treatments. The histogram is shown that fewer mRNAs have altered expression after FT (318 up-regulated and 398 down-regulated in FT4 sample; 221 up-regulated and 384 down-regulated in FT8 sample) compared to chilling stress response (745 up-regulated and 336 down-regulated in CT4 sample; 1315 up-regulated and 824 down-regulated in CT8 sample); the amount of up-regulated mRNAs was less than that of down-regulated ones after FT, while the majority of mRNAs were induced by CT (Fig 1A and S1 Table). Then we made a hierarchical clustering of the DE mRNAs based on the five samples’ log_10_ (RPKM+1), the results showed that the samples could be sorted into three distinct groups based on the temperature of cold treatments (Fig 2A). Taken together, CT and FT had a significant impact on global gene expression profile in tea plants, and different mechanisms may underlie the chilling and freezing response of tea plants. Concurrently, a Venn diagram was used to represent the distribution of DE mRNAs among the four comparisons. We can see that only 200 DE mRNAs overlapped among the four comparisons, which may have some housekeeping functions in both chilling and freezing response of tea plants. In addition, there are 212, 1,131, 159 and 98 mRNAs altered the expression uniquely in CT4, CT8, FT4 and FT8 samples, respectively (Fig 1B).

### Cold-responsive transcription factor genes

**Fig 3:**
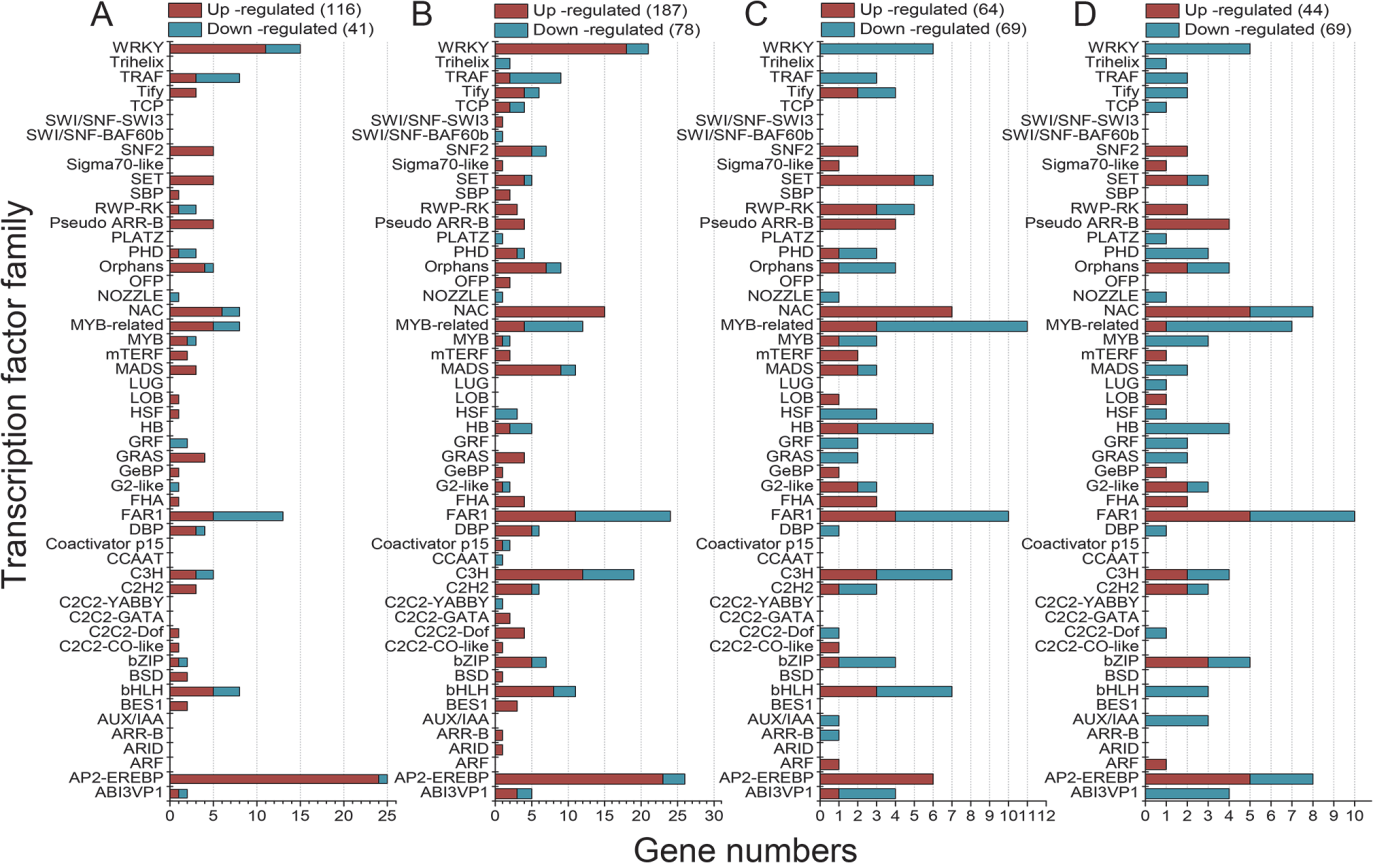
Distribution of differentially expressed transcription factors. The histograms showing the number of up- or down-regulated transcription factors in CT4 (A), CT8 (B), FT4 (C) and FT8 (D) samples, respectively.

A total of 668 transcription factor unigenes belonging to 52 transcription factor families displayed significant difference in expression (Fig 3). We noted that, compared with CT samples (116 up-regulated and 41 down-regulated in CT4 sample; 187 up-regulated and 78 down-regulated in CT8 sample), fewer transcription factor genes were found to have altered expression in FT samples (64 up-regulated and 69 down-regulated in FT4 sample; 44 up-regulated and 69 down-regulated in FT8 sample) and the majority of them were repressed in response to freezing stress. Hence, it can be expected that transcriptional activations are involved in the CT samples while transcriptional repressions are implicated in the FT samples (Fig 3).

### GO and KEGG pathway enrichment analysis of differentially expressed (DE) mRNAs

**Fig 4:**
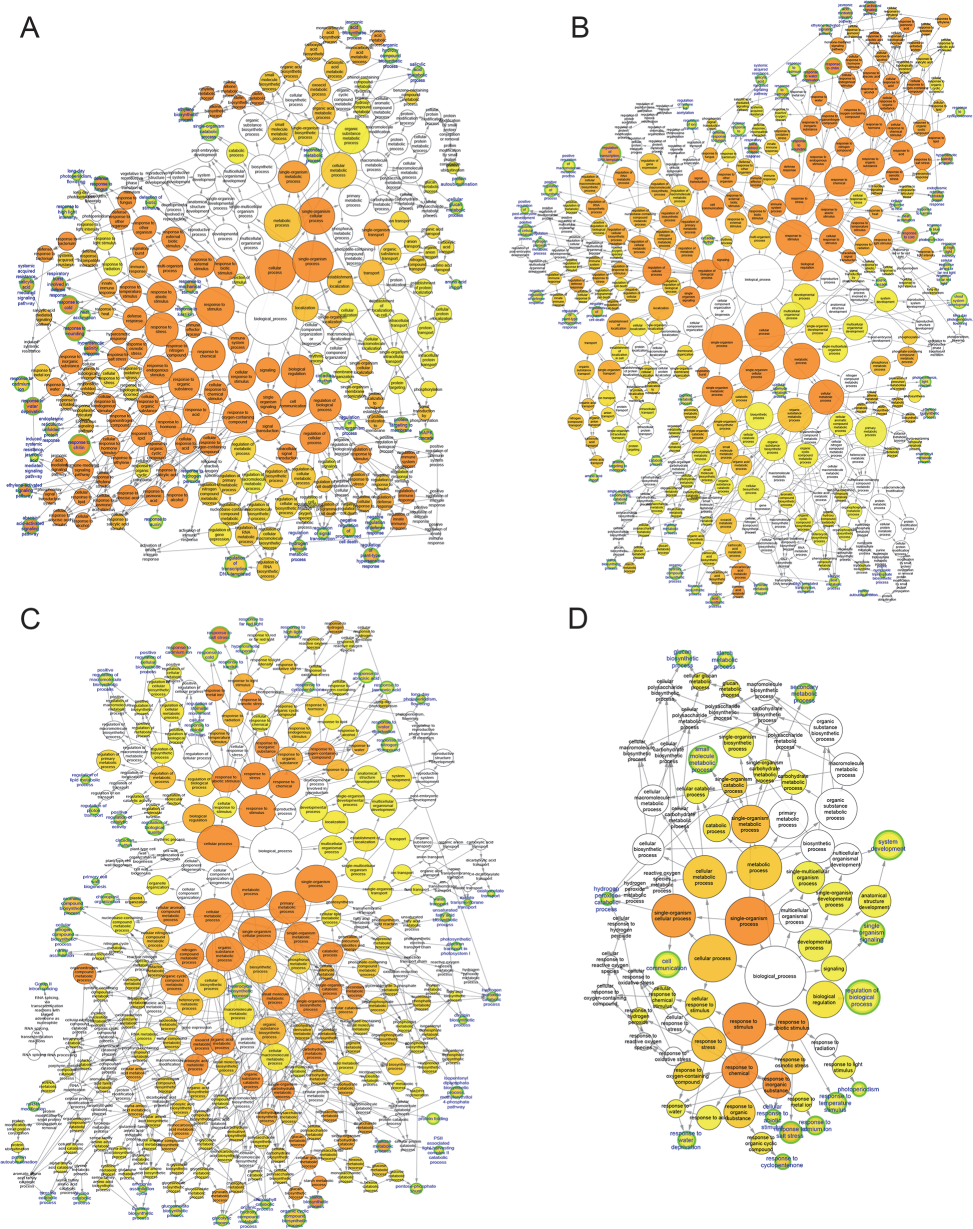
Biological process network of GO term enrichment for differentially expressed (DE) mRNAs. Over-represented GO terms for DE mRNAs in CT4 (A), CT8 (B), FT4 (C) and FT8 (D) samples, respectively. The node size represents the number of genes associated to a given GO term and node filled color reflects the adjusted *P*-value. End nodes were indicated by blue label and green border.

**Fig 5:**
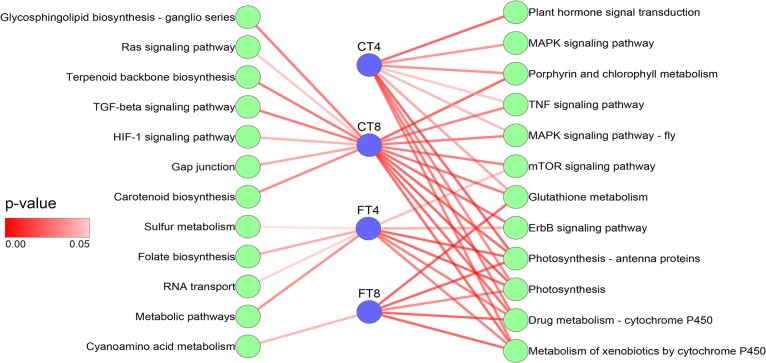
KEGG pathway analysis of differentially expressed (DE) mRNAs. A total of 10, 17, 10 and 6 significantly enriched KEGG pathways were identified in CT4, CT8, FT4 and FT8 samples, respectively. Line color of edge represents the *P*-value of pathway.

To elucidate the correlation between cold (chilling and freezing) response and the biological processes/pathways, functional classification of DE mRNAs was performed using GO term and KEGG pathway enrichment analysis. GO enrichment analysis using DE mRNAs showed that some important GO terms, such as ‘response to cold’, ‘response to karrikin’, and ‘starch biosynthetic process’ were overlapped in both CT and FT groups; but several crucial biological processes related to signal transduction (e.g., ‘MAPK cascade’), response to stimulus (e.g., ‘response to chitin’), hormone-mediated signaling pathway (e.g., ‘abscisic acid-activated signaling pathway’), carbohydrate metabolic process (e.g., ‘glucan biosynthetic process’), and reactive oxygen species (ROS) metabolic process (e.g., ‘hydrogen peroxide catabolic process’), as well as photosynthesis (e.g., ‘photosynthesis, light reaction’) were distinct among treatment groups (Fig 4 and S2 Table). By performing the KEGG pathway analyses, totally 24 pathways that changed significantly (*p* ≤0.05) after CT and FT were identified. Several key pathways, such as ‘Plant hormone signal transduction’ (ko04075), ‘Photosynthesis’ (ko00195), ‘MAPK signaling pathway’ (ko04010), ‘Metabolism of xenobiotics by cytochrome P450’ (ko00980), ‘Glutathione metabolism’ (ko00480), and ‘Starch and sucrose metabolism’ (ko00500) were involved and function in the early response to cold stress (Fig 5 and S3 Table). These results imply that our cold stress treatments were efficient and genes involved in these biological processes and pathways may play crucial roles in cold stress response of tea plants.

### miRNAs regulate expression changes of tea plants in response to cold stress

#### Sequencing and data analysis

To identify cold-responsive miRNAs in tea plants, five small RNA (sRNA) libraries were constructed from tea leaves with or without cold treatment. After removing contaminant reads, 8.8, 7.2, 7.2, 8.0 and 7.7 million clean reads were generated in CK, CT4, CT8, FT4 and FT8 samples, respectively (S4 Table). The majority of the conserved miRNAs start with a 5' terminal uridine residue (S3 Fig), a conserved feature of miRNAs recognized by the AGO1 protein [43]. The length distributions of sRNAs were similar among libraries. The 24 nt sRNAs were the most abundant followed by 21 nt long sequences, which was in agreement with previous reports on *A*.*thaliana* [44], grapevine [45] and rice [46]. Compared with CK group, we observed that the proportion of 24 nt sRNAs decreased in CT8, FT4 and FT8 groups with the 21 nt population had a certain level of increase in CT8 group (Fig 1C).

#### Identification of conserved and novel cold-responsive miRNAs and comparison of miRNA expression level among libraries

**Fig 6:**
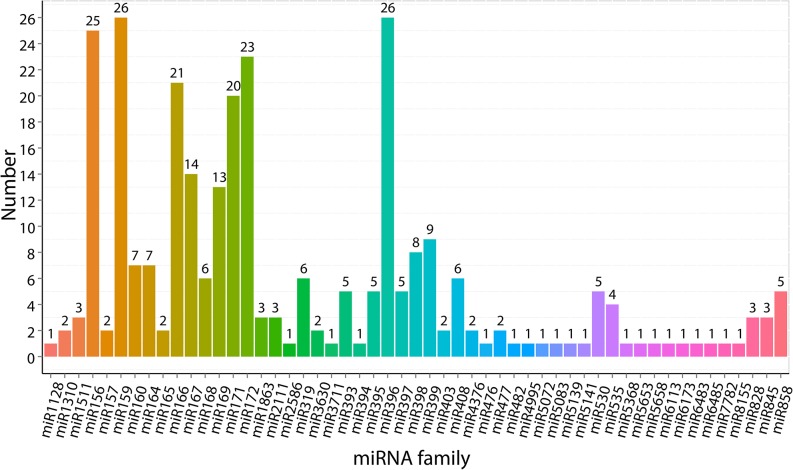
Numbers of miRNA member in each family in *C*. *sinensis*.

In total, we identified 295 conserved miRNAs belonging to 53 miRNA families, and 72 predicted novel miRNAs in the five libraries (S5 Table). The detailed family member numbers of conserved miRNA were summarized in Fig 6. A total of 33 conserved miRNA families contained more than one member. Intriguingly, the abundance of diverse members sequenced from the same or different miRNA families varied drastically, ranging from one to 21,482 times (S5 Table). Differ from the result of DE mRNAs, the number of differentially expressed (DE) miRNAs in FT samples (63 up-regulated and 71 down-regulated in FT4 sample; 55 up-regulated and 68 down-regulated in FT8 sample) were more than that in CT samples (28 up-regulated and 54 down-regulated in CT4 sample; 32 up-regulated and 70 down-regulated in CT8 sample), and the majority of miRNAs were repressed in both CT and FT samples (Fig 1D). This is likely due to the expression of target mRNAs controlled by these repressed miRNAs are turned on in adaptation to cold stress, and more post-transcriptional control by miRNAs might occur in tea plants respond to freezing stress. However, hierarchical clustering of the DE miRNAs based on the five samples’ log_10_ (TPM+1) showed the consistency between miRNAs and mRNAs, that is, five samples were sorted into three distinct groups based on the temperature of cold treatments (Fig 2B). In addition, a total of 233 miRNAs were differentially expressed under cold treatments. Among them, 16, 20, 39 and 28 miRNAs were uniquely differentially expressed in CT4, CT8, FT4 and FT8 samples, respectively. Meanwhile, only 15 DE miRNAs were found to be overlapping among four samples, suggesting the conserved functions of these miRNAs in early cold response of tea plants (Fig 1E).

### Correlation of DE miRNAs and DE mRNAs in response to cold stress

**Fig 7:**
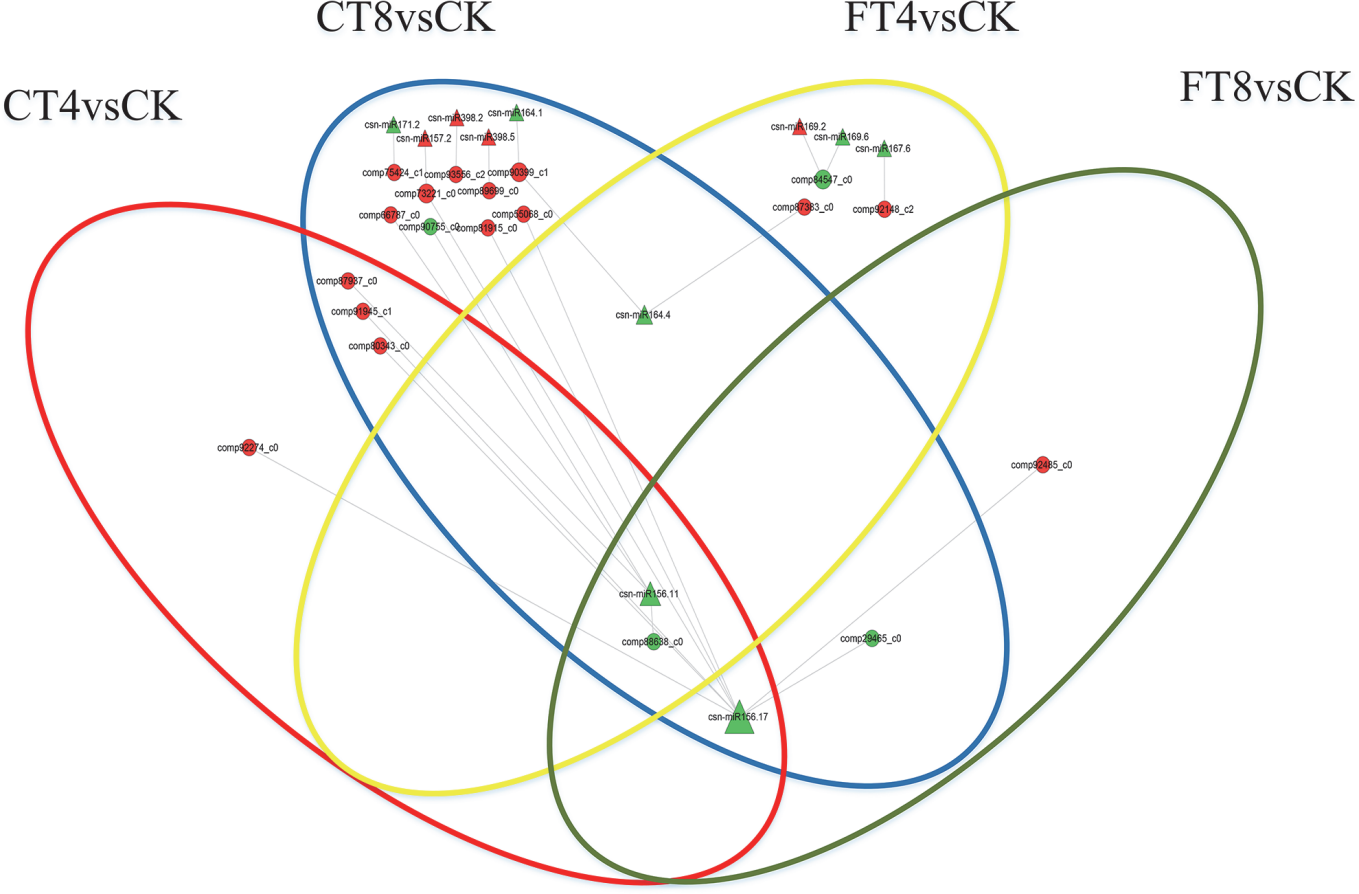
miRNA-mRNA correlation network. Circuits in red, blue, yellow and green ellipse are indicating CT4, CT8, FT4 and FT8 samples, respectively. The triangles and circles in the network are indicating miRNAs and target mRNAs, respectively. Down-regulated mRNAs and miRNAs are shown as green and up-regulated mRNAs and miRNAs are shown as red.

DE miRNAs with its predicted target lists were investigated for cognate mRNA targets in respective DE mRNAs list in order to delineate miRNA-mRNA functional interactions using an in-house R script (S1, S6 and S7 Tables). There were 22 miRNA-mRNA interactions among treatment groups were identified, with the involvement of 11 DE miRNAs (csn-miR156.17, csn-miR156.11, csn-miR157.2, csn-miR164.1, csn-miR164.4, csn-miR171.2, csn-miR398.2, csn-miR398.5, csn-miR167.6, csn-miR169.2 and csn-miR169.6) and 19 DE mRNAs (comp80343_c0, comp91945_c1, comp92274_c0, comp87937_c0, comp88638_c0, comp29465_c0, comp55068_c0, comp73221_c0, comp81915_c0, comp90399_c1, comp75424_c1, comp93556_c2, comp89699_c0, comp66787_c0, comp90755_c0, comp87383_c0, comp92148_c2, comp84547_c0 and comp92485_c0) in total. Given that miRNAs negatively regulate the expression of their target mRNAs by target RNA cleavage, the expression patterns of miRNA target genes generally show an inverse correlation with those of miRNAs. Therefore, for the majority of cases that involve target cleavage, the simple expectation is that, when miRNAs are induced by cold stress, their target mRNAs are reduced and vice versa. Whereas, our study describes here the 7 significantly down-regulated miRNAs (i.e., csn-miR164.4, csn-miR164.1, csn-miR171.2, csn-miR156.11, csn-miR169.6, csn-miR167.6 and csn-miR156.17) and 4 significantly up-regulated DE miRNAs (i.e., csn-miR157.2, csn-miR398.5, csn-miR398.2 and csn-miR169.2) under cold treatments showed both negative and positive correlation with target mRNAs (Fig 7 and S8 Table). From Fig 7, we can see that a single miRNA can regulate multiple target mRNAs and vice versa. The miRNA-mRNA regulatory network is more complexity than previously thought, especially for complicated the different group of mRNAs involved in different treatment groups, regulated by different miRNAs.

Functional classification of the deregulated target mRNAs suggested multiple biological roles. The GO annotation of 15 target mRNAs (comp80343_c0, comp91945_c1, comp92274_c0, comp87937_c0, comp88638_c0, comp29465_c0, comp55068_c0, comp73221_c0, comp81915_c0, comp90399_c1, comp75424_c1, comp93556_c2, comp89699_c0, comp66787_c0 and comp90755_c0) respond to CT involved in ‘carbohydrate metabolic process’, ‘protein phosphorylation’ and ‘regulation of transcription, DNA-dependent’; as per the KEGG classification, ‘glycan biosynthesis and metabolism’, ‘carbohydrate metabolism process’ and ‘transport and catabolism’ pathways were included. In addition, the 6 target mRNAs (comp87383_c0, comp92148_c2, comp84547_c0, comp88638_c0, comp29465_c0 and comp92485_c0) respond to FT were annotated as ‘phosphorylation’, ‘signal transduction’, ‘phosphorelay signal transduction system’, ‘auxin mediated signaling pathway’, ‘regulation of transcription, DNA-dependent’, ‘carbohydrate metabolic’ and ‘flower development’; the pathways included ‘glycan biosynthesis and metabolism’, ‘carbohydrate metabolism process’, ‘transport and catabolism’ and ‘signal transduction’ (S8 Table).

### qRT-PCR validation of significant DE miRNAs and mRNAs

**Fig 8:**
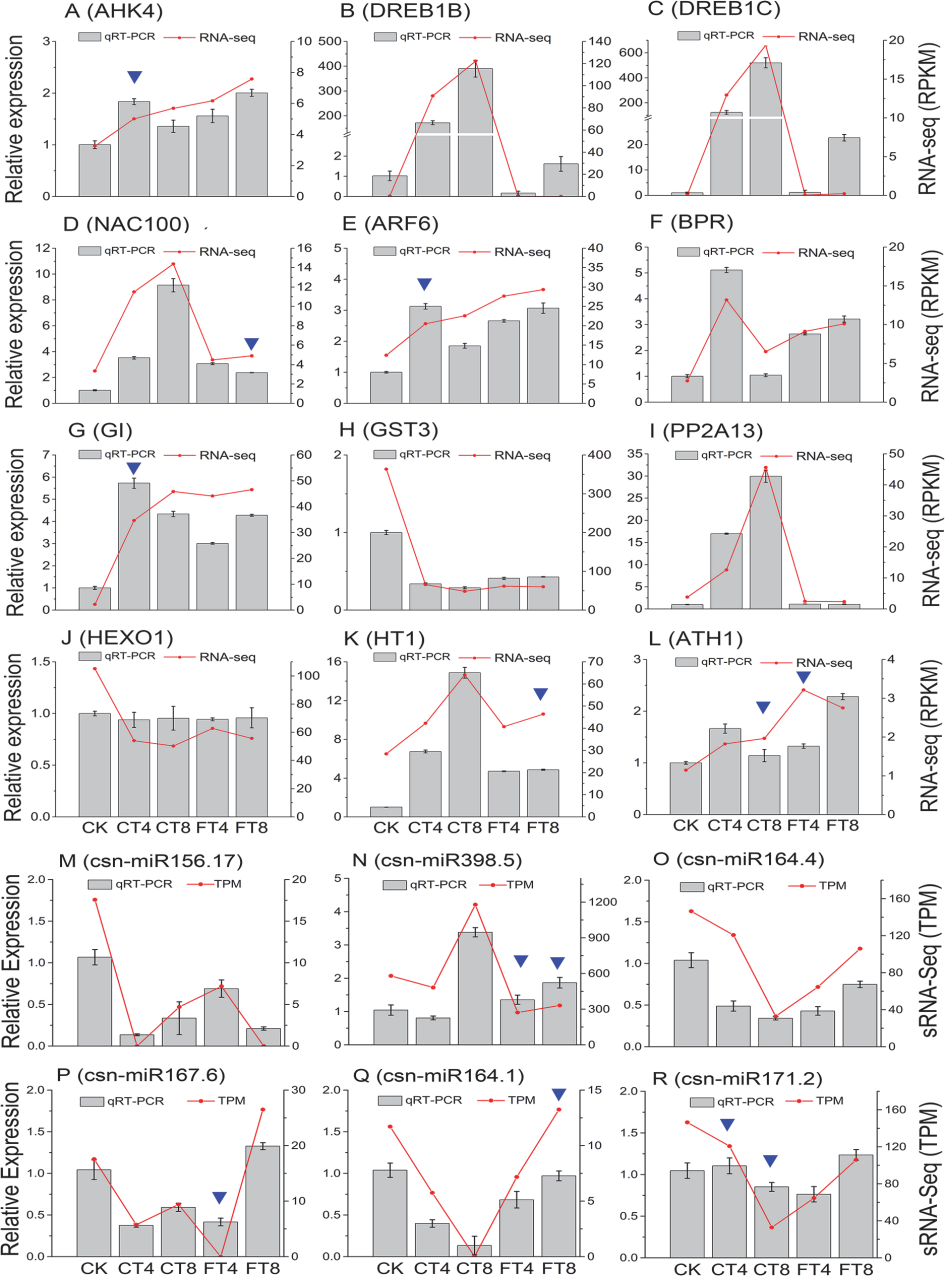
qRT-PCR validation for DE miRNAs and mRNAs. Twelve DE mRNA and six DE miRNA were selected for the quantitative RT-PCR analysis, including AHK4 (A), DREB1B (B), DREB1C (C), NAC100 (D), ARF6 (E), BPR (F), GI (G), GST3 (H), PP2A13 (I), HEXO1 (J), HT1 (K), ATH1 (L), csn-miR156.17(M), csn-miR398.5(N), csn-miR164.4(O), csn-miR167.6(P), csn-miR164.1(Q) and csn-miR171.2(R). The GADPH gene and 5.8S rRNA were chosen as the endogenous control for mRNA and miRNA qRT-PCR, respectively.

To further test the reliability of our RNA-Seq and sRNA-Seq data, qRT-PCR analysis was performed on 6 DE miRNAs (csn-miR156.17, csn-miR398.5, csn-miR164.4, csn-miR167.6, csn-miR164.1 and csn-miR171.2) among the miRNA-mRNA interaction network, and 12 DE mRNAs among treatment groups. Twelve DE mRNAs were manually selected as representatives for their potential roles in cold response according to their annotations and their potential relationship with cold-responsive miRNAs. These genes encode DREB1B/C (Dehydration-responsive element-binding protein 1B/C), NAC100 (NAC domain-containing protein 100) and ARF6 (Auxin response factor 6) TFs, AHK4 (Histidine kinase 4), BPR (Beta-primeverosidase), GI (Protein GIGANTEA), GST3 (Glutathione S-transferase 3), PP2A13 (F-box protein PP2-A13), HEXO1 (Beta-hexosaminidase 1), HT1 (Serine/threonine-protein kinase HT1) and ATH1 (Homeobox protein ATH1) (Fig 8). The results of qRT-PCR revealed that, most of these mRNAs/miRNAs shared similar expression tendency with those from RNA-Seq/sRNA-Seq data (RPKM/TPM-based expression values), while discrepancies were also found in some treatment groups, and potential discrepancies between qRT-PCR and RNA-Seq/sRNA-Seq results were indicated as blue triangles in Fig 8. Although there were some quantitative differences between the two analytical platforms, the similarities between the RNA-Seq data and the qRT-PCR suggest that the RNA-Seq data are reproducible and reliable (Fig 8).

## Discussion

The findings discussed here reveal the first detailed snapshot of parallel mRNA and miRNA expression levels in tea plants under early cold (chilling and freezing) stress. The RNA-Seq, DGE and sRNA-Seq approaches enabled providing a global transcriptome and miRNome picture and identifying those mechanisms related to chilling and freezing response in tea plants. We performed an integrative analysis of these data and obtained the complete set of early cold-responsive miRNAs/mRNAs, their interactions and dynamics in the biological process/pathway and the differences in biological process/pathway within and between different cold treatments, which could help us tell the differences between chilling and freezing response mechanisms in tea plants.

### Expression and regulatory patterns of cold-responsive miRNAs/mRNAs in tea plants

Some studies have demonstrated that the stress-responsive miRNA-mRNA regulatory network includes both the coherent and incoherent regulatory patterns [38]. In this work, we attempt to construct the miRNA-mRNA regulatory network according to the DE miRNAs and DE mRNAs datasets, sample categories and miRNA-targeting information. Finally, 22 miRNA-mRNA pairs, including both positive and negative correlation were identified.

As shown in Fig 7, the miRNA-mRNA regulatory network is more subtle and complex than we had believed, especially for selective and complex correlation of miRNAs and target mRNAs. For instance, a single miRNA can target multiple mRNAs (e.g., comp29465_c0, comp55068_c0, comp73221_c0, comp80343_c0, comp81915_c0 and comp91945_c1 can be simultaneously regulated by csn-miR156.17 in CT8 sample). Further, a specific miRNA may regulate certain specific target mRNAs and thus contribute to the response of specific period or temperature of cold treatments (e.g., comp92274_c0, comp55068_c0 and comp92485_c0 can be regulated by csn-miR156.17 in CT4, CT8 and FT8 samples, respectively). Additionally, common target mRNAs might be regulated by multiple cold-responsive miRNAs, even between up- and down-regulated miRNAs (e.g., comp73221_c0 can be negatively regulated by down-regulated csn-miR156.17 and up-regulated csn-miR157.2 in CT8 sample), suggesting that these miRNAs are responsible for both switch on/off and fine-tune target mRNA expression under cold-stress-conditions. In many cases, the expression pattern of the miRNA and its targets show an opposite trend. Although this negative correlation between miRNA and their target mRNAs is often considered proof of miRNA targeting, there are also reported cases of positive correlation at the expression level of miRNA and their target mRNAs [47]. In *Arabidopsis*, a well-characterized example is the case of miR168 targeting of AGO1. They co-expressed everywhere to maintain a proper balance between the miRNA and AGO1 expression levels when needed, which suggesting the involvement of fine-tuned mechanisms to modulate gene expression through a miRNA mediated feedback loop [48]. Moreover, miRNAs from the same miRNA families displayed different expression patterns (e.g., down-regulated csn-miR169.6 and up-regulated csn-miR169.2 in FT4 sample), implying their different roles in regulating expressions of the cold-responsive mRNAs or that their target mRNAs are diversified (Fig 7). This is consistent with the results obtained from cold-stressed populus [49] and peach [50]. These cold-responsive miRNA members from the same family may be further differentiated by their spatio-temporal response to cold stress. Therefore, the functions of plant miRNAs can be dissimilar even if they share a high degree of sequence similarity. Additionally, it has been demonstrated that miRNAs may indirectly interact with mRNAs through other TFs, miRNAs or endo-siRNAs (e.g. ta-siRNAs) besides direct base pairing [36]. Consequently, indirect mRNA modulatory effects of miRNAs to decrease or increase target mRNAs may be frequent or even greatly outnumber direct target suppressions [38]. Hence, miRNA-mRNA showing both the coherent and incoherent regulatory patterns is not unusual. Further investigation to unravel the TFs and relevant genes responsible for regulating the miRNA expression may shed light on the complex miRNA-mRNA interplay observed in various studies.

### GO and pathways involved in response to chilling and freezing stress of *C*. *sinensis*

Functional and pathway assignments of the DE mRNAs and DE miRNAs-mediated targets using GO and KEGG classification revealed numerous hormonal, metabolic, physiological, and developmental responses, which may played vital roles in response to chilling and freezing stress in tea plants. These included alterations in (i) photosynthesis, (ii) protein phosphorylation, (iii) protein autoubiquitination, (iv) transcription factors, (v) carbohydrate metabolism and (vi) plant hormone signal transduction (Figs 4 and 5; S2, S3 and S8 Tables). Photosynthesis is usually the first to be influenced by changing temperatures. In barley and *Arabidopsis*, it has been reported that photosynthetic activity plays an important role in plant cold resistance [51, 52]. In our study, ‘Photosynthesis’ and ‘Photosynthesis—antenna proteins’ pathway was significantly enriched in both CT and FT samples, suggesting a role in the early response to cold stress (Fig 5 and S3 Table). Detailed information of ‘Photosynthesis’ pathway revealed that several photosynthesis related genes were rapidly induced in CT4 and FT4 groups; whereas most photosynthesis related genes were down-regulated or unchanged in CT8 and FT8 samples. Moreover, enrichment of GO terms such as ‘response to hydrogen peroxide’ (CT samples) and ‘hydrogen peroxide catabolic process’ (FT samples), and ‘Glutathione metabolism’ pathway (CT and FT samples) implied that light-induced reactive oxygen species (ROS) production might occur (Figs 4 and 5; S2 and S3 Tables). Furthermore, the ‘Metabolism of xenobiotics by cytochrome P450’ was identified as prominently enriched pathway in both CT and FT samples (Fig 5 and S3 Table). The members of the P450 superfamily were known to play a role in abiotic stress via their transcriptional up-regulation in response to ROS, which have a dual role in biotic and abiotic stress response of plants [53]. While ROS are thought to be one of the main causes of stress-induced damage to proteins, lipids and nucleic acids by oxidative reactions, ROS can also play a key role in mediating stress-related signal transduction events that help the plant in mounting a quick and credible response to mitigate cold-induced damage [54]. In a recent study conducted on poplar, the amount of ROS was produced early in the chilling response resulting in photosynthesis being inhibited and producing a survival advantage [55]. Taken together, our results were consistent with previous reports that the repression of some genes involved in photosynthesis pathway and the accumulation of ROS may be considered as a result of cold shock response, which could have active functions such as the protection from the photodamage in tea plants.

Post-translational modifications are reversible, constituting very versatile mechanisms that allow plants to finely adjust their responses to cold stress [21]. The disruption of phosphorylation has a significant impact on the ability of plants to acclimate to cold stress [56]. Several protein kinases belonging to diverse families have shown to be involved in the perception of environmental signals, such as histidine kinases (HKs), serine/threonine-protein kinase and mitogen-activated protein kinases (MAPKs). HKs are the major signaling elements and are involved in the multistep two-component signaling system for perception and transduction of cold stress signaling [57]. In chrysanthemum, both the serine/threonine-protein kinases and MAPK pathway are involved in the processes of cold acclimation and chilling/freezing response [58]. Here, csn-miR156.17 was significantly down-regulated (5.2-fold) with its target gene AHK4 was up-regulated (1.1-fold) in FT8 sample. Therefore, it can be expected that the two-component signaling pathways might be activated by repressing the csn-miR156.17 in tea plants and so enhance the adaptability to freezing stress (S8 Table). Although csn-miR398.5 functions as a repressor, we showed here that it is co-expressed in CT8 sample with its target gene HT1 (Serine/threonine-protein kinase HT1) in which miRNAs fine-tune or maintain transcriptionally established gene expression patterns under chilling stress (S8 Table). We confirmed by qRT-PCR that AHK4 and HT1 expression is induced in both, which indicates that protein kinases as well as their regulating miRNAs play an important role in the early chilling/freezing response of tea plants (Fig 8A,8K,8M and 8N). MAPKs are implicated in the signaling of most plant hormones and developmental processes, and also acted as the converging points of various abiotic stress signalling pathways. [59, 60]. As indicated in Fig 5, ‘MAPK signaling pathway’ was significantly enriched in both CT4 and CT8 samples (S3 Table). Furthermore, in the GO cluster of ‘response to stimulus’, the network patterns of treatment groups showed that a wide range of stress responses were aroused by cold stress in both CT and FT samples (e.g., 'response to water deprivation' and 'response to high light intensity'), which were probably due to the contribution of MAPKs and other cross-tolerance mechanisms of tea plants (Fig 4 and S2 Table). MAPKs were known to be activated by cold-induced ROS, which further suggesting the generation of ROS in the early cold response [61]. Additionally, the effects of ROS on components of the MAPK cascades have shown to result in the indirect activation of transcription factors [62].

Transcription factors (TFs) play a crucial role in plant development and stress response [63]. As shown in Fig 3, at least five TF families have been reported to be linked to cold stress resistance in plants, including NAC, MYB, bHLH, WRKY, and AP2-EREBP [19, 20]. One group of well-studied transcription factors involved in cold responses is the AP2-EREBP family members, which have been subdivided into four major subfamilies: the DREB/CBF, AP2, ERF and RAV subfamilies [64]. Of these, the DREB/CBF subfamily has been reported to play a major role in the early stages of the cold response [65], as evidenced by studies in numerous species such as *Anthurium* [40] and rice [66], as well as tea plant [2]. This family contains 24, 23, 6 and 5 up-regulated members in the CT4, CT8, FT4 and FT8 groups, respectively (Fig 3). Surprisingly, none of the DREB1s/CBFs (DREB1b/CBF1, DREB1c /CBF2 and DREB1a/CBF3) homologous gene was found in the FT4 and FT8 groups though CBF1 (comp79005_c0) and CBF2 (comp73809_c0) were both significantly up-regulated over 7-fold in the CT4 and CT8 groups, suggesting a key role of the DREB1/CBF TFs in tea plants’ chilling but not freezing stress perception/response. Their cold-induced expression evident from the RNA-seq was further confirmed by qRT-PCR (Fig 8B and 8C). Some RAP homologs (e.g. comp89295_c0) and ERF homologs (e.g. comp98269_c0), which were seldom studied in cold response were mostly up-regulated in all treatment groups, indicating their potential role in cold response (S1 Table).

The majority of WRKY family members are known to be responsive to various abiotic stresses and function as regulators of plant basal disease resistance [67, 68]. A growing body of evidence has shown that WRKY proteins are involved in responses to cold stress [20, 40, 67]. It is notable that 11 out of 15 and 18 out of 21 WRKY family members in CT4 and CT8 groups were up-regulated in response to chilling stress, respectively. Whereas all the WRKY family members were found down-regulated in FT groups (Fig 3). The results not only suggest that tea plants may respond to chilling and freezing stress with different strategies but also indicate a crosstalk between plant-pathogen interaction and cold stress response may occur.

A comprehensive expression analysis of MYB superfamily showed that most of MYB genes are responsive to various phytohormones and stress conditions [69]. The majority of bHLH TFs show late down-regulation under cold stress [70]. Furthermore, some reports suggest that MYB and bHLH TFs often interact with each other to control transcription [71]. In the present study, most of the MYB or MYB-related family members showed down-regulation in CT8, FT4 and FT8 groups. The bHLH family members were mostly induced in CT groups, while the majority of which were repressed in FT groups (Fig 3). Therefore, based on our analysis, we speculated that distinct ratios of these partner factors may be required in response to different cold conditions.

In addition to the above-mentioned TFs, the plant-specific NAC family members have been implicated in plant development (e.g., seed development, embryo development, shoot apical meristem formation, and leaf senescence) [72], as well as multiple abiotic and biotic stress responses (e.g., drought, salinity, cold and virus infections) [73]. We have identified 6, 15, 7 and 5 NAC TF genes were up-regulated in response to cold stress, while only 2, 0, 0 and 3 members of NAC family genes showed repressed expression in CT4, CT8, FT4 and FT8 groups, respectively (Fig 3). The over-representation of this TF family may indicate their importance in tea plants’ cold response. Some studies have suggested that the miR164 family members guide the cleavage of the NAC genes to modulate developmental processes, as well as abiotic stress [74, 75]. To date, the functions of the miR164 and its targeted NAC genes in tea plants are poorly deciphered. Our results show that down-regulated csn-miR164.1/4 (2.0-fold) and its targeted NAC gene (comp90399_c1, up-regulated by 2.0-fold) in CT8 samples may be positive regulators of chilling response in tea plants, apart from their reported roles in development (S8 Table, Figs 3, 7 and 8D, 8O and 8Q). However, in *P*. *trichocarpa*, miR164 was reported to be up-regulated within 8 h of the cold stress treatment, and then showed a decrease trend [49]. Such discrepancies on the expression pattern of miRNAs may be due to variations in either technical reasons or experimental conditions. Nevertheless, it is also possible that these miRNAs play a species-specific role in cold responses. Besides, miR167 is reported to be involved in regulating the auxin signal by cleavage of two auxin response factors (i.e., ARF6 and ARF8) [76]. The plant hormone auxin regulates various growth and developmental processes as well as responses to environmental stress conditions [77, 78]. In our study, cold-induced increases of ARF6 (comp92148_c2, 1.1-fold) were modulated by down-regulation (5.1-fold) of csn-miR167.6 in FT4 group, indicating that csn-miR167.6 might play a potential role in freezing stress response by affecting the auxin signaling pathway in tea plants (S8 Table, Figs 3, 7 and 8E and 8P).

Taken together, these conserved targets of known miRNA families which code for transcription factors or kinases known to control key steps in plant development, such as miR156-SPL, miR164-NAC, miR167-ARF6, miR398-HT1 and miR156-AHK4, have also been found to be regulated by environmental stresses and thus may serve to integrate stress responses/memory with development. This kind of regulatory cascade is especially significant for early cold-response by modulating the expression of stress-regulated genes among multiple stress perception and signalling pathways in plants. In addition, most of them were also experimentally validated by using either RLM-RACE (a modified RNA ligase mediated random amplification of cDNA ends) or degradome sequencing in previous studies [79–81], which further validate the robustness of our method to predict rather reliable miRNA-mRNA duplexes in the specific biological context.

**Fig 9:**
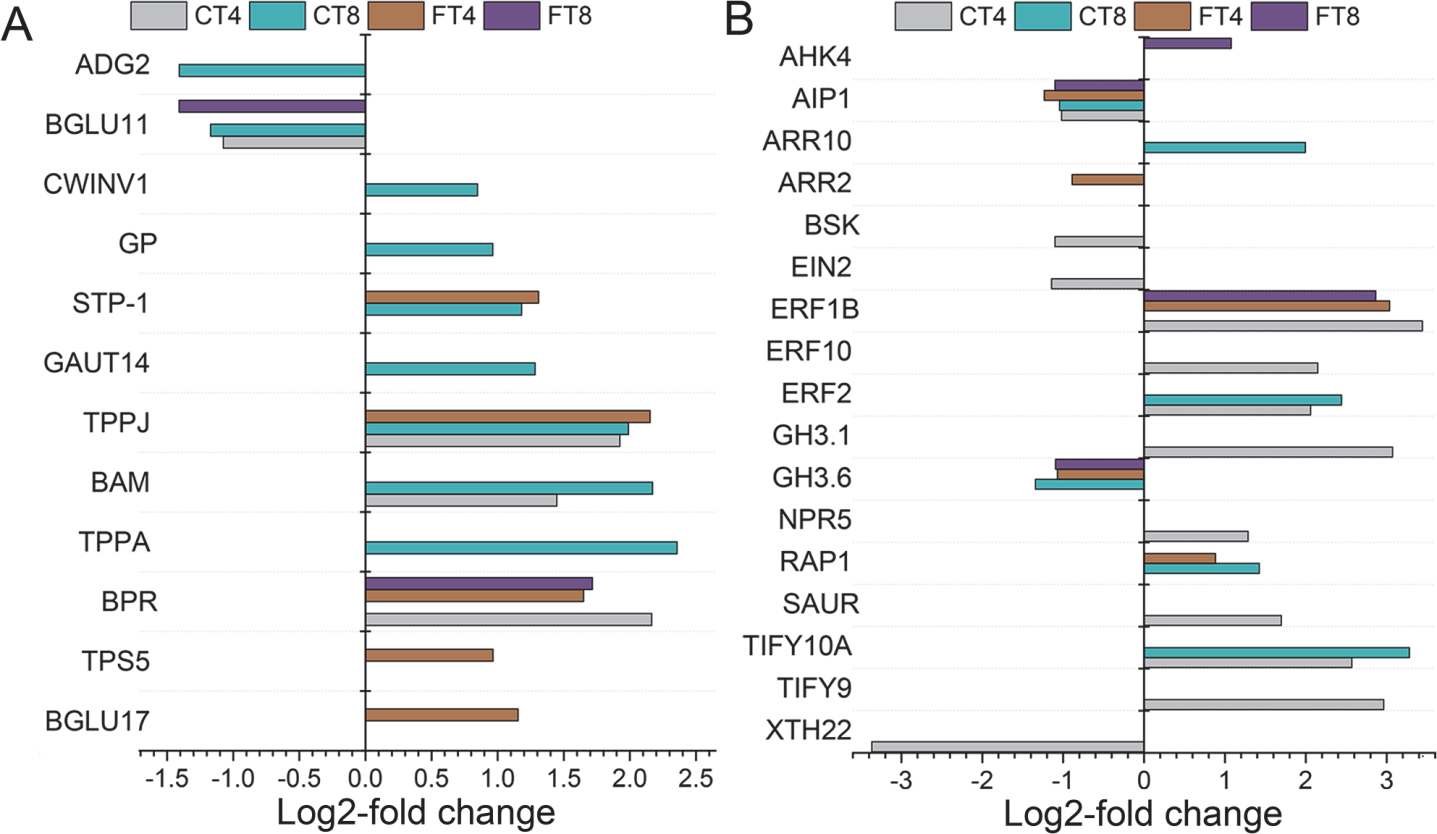
Differential expression of carbohydrate metabolism and plant hormone signal transduction related genes. The histograms showing the number of up- or down-regulated carbohydrate metabolism (A) and plant hormone signal transduction (B) related genes in CT4, CT8, FT4 and FT8 samples, respectively.

Cold stress also induce the rapid accumulation of soluble carbohydrates in plants. As indicated in Fig 9A, the enzymes up-regulated in CT samples involved in starch turnover were β-amylase (BAM) and glucan phosphorylase (GP), each has been reported to be induced by abiotic stress [82, 83]. Beta-glucosidases (BGLUs) are implicated in the timely response to abiotic and biotic stresses in plant through activation of defense compounds and plant hormones [84]. We observed that BGLU17 was uniquely induced in FT4 group, whereas BGLU11 was repressed in CT4, CT8 and FT8 groups in response to cold stress. In addition, the expression levels of crucial enzymes (TPPA/J, trehalose-phosphate phosphatase A/J and TPS5, alpha, alpha-trehalose-phosphate synthase [UDP-forming] 5) for synthesizing trehalose increased under cold stress. Among them, TPS5 and TPPA were induced in FT4 and CT8 groups, respectively; TPPJ displayed an increase in CT4, CT8 and FT4 groups, indicating the important role of trehalose in tea plants’ cold resistance. Intriguingly, we notice that β-primeverosidase (BPR), a key enzyme involved in the formation of tea aroma were identified as being quickly induced in CT4, FT4 and FT8 groups, which may be explained by the fact that the BPR hydrolyzes β-primeverosides to liberate a primeverose unit and aglycons such as methyl salicylate to induces various defense responses in response to biotic and/or abiotic stress [85]. Additional evidence for cold-induced expression changes of BPR among treatment groups were provided by qRT-PCR analysis (Fig 8F). Thus, further investigation should be conducted to clarify the correlation between tea leaf BPR/tea quality (aroma) and cold stress, which can shed light on and enhance our knowledge of the function of BPR in tea plants.

Phytohormones are essential for the ability of plants to adapt to abiotic stresses by mediating a wide range of adaptive responses [4]. ‘Plant hormone signal transduction’ pathway comprised 12, 6, 5 and 4 genes of pathway-hit genes in CT4, CT8, FT4 and FT8 groups, respectively (Fig 9B). In this pathway, the transcripts of TIFY9/10A (Protein TIFY 9/10A), SAUR (auxin-responsive protein), NPR5 (nonexpressor of pathogenesis-related gene 5), GH3.1 (Indole-3-acetic acid-amido synthetase GH3.1), ERF2/10 (Ethylene-responsive transcription factor 2/10), and ARR10 (Two-component response regulator ARR10) were determined to be up-regulated, while the transcripts of XTH22 (Xyloglucan endotransglucosylase/hydrolase protein 22), EIN2 (Ethylene-insensitive protein 2), BSK (BR-signaling kinase) were identified as being down-regulated in CT samples. Additionally, we found that the expression levels of RAP1 (Transcription factor MYC2) and ERF1B (Ethylene responsive element binding protein) transcripts were determined to be up-regulated, while the transcripts of GH3.6 (Indole-3-acetic acid-amido synthetase GH3.6) and AIP1 (Protein phosphatase 2C 3) were detected to be down-regulated in both CT and FT samples. Notably, the transcripts of ARR2 (Two-component response regulator ARR2) and AHK4 were found to be down- and up-regulated exclusively by freezing exposure, respectively (Fig 9B). Their homologous genes were identified as being involved in jasmonic acid (JA), auxin (IAA), salicylic acid (SA), abscisic acid (ABA), cytokinin (CK), brassinosteroid (BR), and ethylene (ETH) mediate hormone signal transduction in plants [86–90]. In addition to ABA and ETH, it is now known that various phytohormones such as JA, SA, and IAA crosstalk extensively to regulate practically all aspects of plant stress responses [4]. In addition, the biological process ‘response to karrikin’ was found to be enriched in both CT and FT groups (S2 Table and Fig 4). Furthermore, the up-regulation of the comp74326_c0 unigene (GI) and the down-regulation of the comp66051_c1 unigene (GST3) involved in ‘response to karrikin’ were also validated by qRT-PCR analysis (Fig 8G and 8H). Karrikins are a group of recently discovered plant growth regulators identified in smoke from wildfires [91]. In addition to alleviating dormancy, crosstalk with other signalling systems including hormones, light and abiotic stress has also been identified [91–93]. For example, the GO term ‘response to karrikin’ was previously found to be enriched after chilling stress in both *Arabidopsis* and *C*. *bungeana* by transcriptome analysis [94]. It was previously shown that smoke/karrikin could enhanced the ubiquitination of proteins and activated protein degradation related genes, and the occurrence of stress-related genes induced by smoke/karrikin were robust and are extensive among the responses, especially cold, heat and biotic stresses in the early post-germination phase [92]. Protein autoubiquitination may play similar roles as protein phosphorylation to be a rapid pathway to get tolerance in plant responses to chilling stress and have important roles in plant cold acclimation [95]. It is of great interest that the processe “protein autoubiquitination” was over-represented in CT4, CT8 and FT4 groups, which may interferes with the karrikin-related signal transduction (S2 Table). It is highly possible that the karrikin ‘signal’ is perceived by a receptor that is shared with the signal transduction system implied in perceiving environmental cues (especially stresses and light), and thus act as a potential hardening factor to increase cold tolerance of tea plants. Therefore, it is also worthwhile and necessary to further clarify correlation between the karrikins and the cold response mechanisms in plants. In the near future, extensive transcriptome and post-transcriptome analysis of tea plants treated with smoke/karrikin and exposed to different stresses will be carried out in our laboratory to draw the map of possible interactions of smoke and stress related pathways.

## Conclusions

To the best of our knowledge, this study is the first exploration to simultaneously characterize the transcriptome and miRNome of tea plants respond to chilling and freezing stress. A large number of mRNAs and miRNAs from tea plants involved in diverse biological processes/pathways were identified. Furthermore, the comparison of several key pathways (e.g., ‘Photosynthesis’), GO terms (e.g., ‘response to karrikin’) and transcription factors (TFs, e.g., DREB1b/CBF1) provided informative results, which could help us tell the differences between chilling and freezing response mechanisms in tea plants. Compared with short-term chilling treatment, fewer genes were found to be differentially expressed after transient freezing treatment. Whereas more post-transcriptional control by miRNAs might occur in tea plants respond to freezing stress. Moreover, we highlighted the diverse regulatory patterns of target gene expression by early cold-responsive miRNAs. In addition to coherent modulation by miRNAs, incoherent correlations between miRNAs and their target mRNAs provide another layer of gene regulatory networks in a cold-responsive pathway. Interestingly, we found that karrikins, a new group of plant growth regulators, might act as a potential hardening factor to increase cold tolerance of tea plants. Furthermore, our result suggest that β-primeverosidase, a key enzyme functionally relevant with the formation of tea aroma might be involved in both chilling and freezing response of tea plants. Hence, it is of paramount interest to draw the map of possible interactions of smoke/karrikin, BPR and stress related pathways by conducting extensive transcriptome and post-transcriptome analysis of tea plants treated with smoke/karrikin and different stresses.

Despite the apparent conceptual simplicity of the construction of miRNA-mRNA regulatory networks by integrative analysis of mRNA and miRNA data, the coherent and incoherent relationships between miRNAs and their target mRNAs are complex and dynamic; and the miRNA, TF or endo-siRNA (e.g. ta-siRNAs) mediated interactions are not yet well-characterized. Thus, the combination of multilevel high throughput deep sequencing datasets [e.g., mRNA and miRNA expression profiling data, ChIP-Seq (chromatin immunoprecipitation with sequencing) data of transcription factors and degradome sequencing data] with bioinformatics analysis could serve as a powerful tool to build better network-based molecular models to predict, test and identify robust cold-stress-responsive miRNA-mRNA pairs. Moreover, systemic analysis of miRNA-mRNA regulatory network from diverse tissues or cell types and during time courses is required, which could provide a better understanding of gene regulatory network in stress response pathways. Taken together, although this study cannot completely account for cold tolerance in tea plants, we provided a molecular basis elucidating the mechanism of tea plants respond to early chilling and freezing stress and provide a good case study to joint analysis of mRNA and its regulation by miRNA in plants.

## Materials and Methods

### Plant material, growth conditions and treatments

The tea plant cultivar ‘*Camellia sinensis* (L.) O. Kuntze cv. *Longjing 43*’ was planted in the Germplasm of Qingdao Tea Repository at the Tea Research Institute, Qinddao Agricultural University (TRI, QAU). All the two-year-old potted tea plants were housed at 25°C under a 16 h light photoperiod (110–150 μmol·m^-2^·s^-1^) for 7 days in the greenhouse prior to cold-stress treatment. Plants with a uniform growth status were transferred to a chamber for cold-stress treatment at 4 or -5° under weak light condition (45–55 μmol·m^-2^·s-^1^). The fully expanded leaves at the third leaf were harvested after 4- and 8-h of cold treatments, untreated leaves were harvested as controls (0 h). More than 5 plants were harvested and pooled for each cold treatments, and the collection was repeated 2 times as biological replicates. Plant materials were quick frozen in liquid nitrogen upon harvest and kept at -80°C until RNA extraction.

### RNA extraction

The total RNA from the tea leaves was extracted using TRIzol reagent (Invitrogen, Burlington, ON, Canada). The quality, purity, concentration and integrity of the total RNA was checked using 1% agarose gel electrophoresis, NanoDrop Photometer Spectrophotometer (IMPLEN, Westlake Village, CA, USA), Qubit RNA Assay Kit in Qubit 2.0 Flurometer (Life Technologies, Carlsbad, CA, USA), and RNA Nano 6000 Assay Kit of the Bioanalyser 2100 system (Agilent Technologies, Santa Clara, CA, USA), respectively.

### Illumina sequencing and *de novo* assembly

For transcriptome and DGE library construction, 3 μg of total RNA per sample was used for the RNA sample preparations. The library for sequencing was generated using Illumina TruSeq RNA Sample Preparation Kit (Illumina, San Diego, USA). After cluster generation, the library preparations were sequenced on an Illumina Hiseq 2000 platform, 100 bp paired-end reads and 50 bp single-end reads were generated from transcriptome and DGE sequencing, respectively. In total 138,748,418 clean data were obtained by discarding the low quality raw reads from the sequencing machines and used for *de novo* assembly of the unigenes by the Trinity program (release 20121005) [96].

### Reads mapping to the reference transcriptome and differential expression analysis

Clean reads and count number of five DGE libraries (CK, CT4, FT4, CT8 and FT8) were assessed and summarized using custom Bioperl scripts. All clean reads were mapped back onto the assembled transcriptome generated by RNA-Seq. Differential expression analysis was performed using the DESeq R package (1.10.1). The resulting *P*-values were adjusted using the Benjamini and Hochberg’s method [97]. Genes with an adjusted *P*-value <0.05 found by DESeq were assigned as differentially expressed.

### Functional annotation and characterization of unigenes and DE mRNAs

Gene function was annotated based on the following databases: Nr (NCBI non-redundant protein sequences); Nt (NCBI non-redundant nucleotide sequences); Swiss-Prot (A manually annotated and reviewed protein sequence database); Pfam (Protein family); GO (Gene Ontology); KO (KEGG Ortholog database); KOG (euKaryotic Ortholog Groups). Plant Transcription Factor Database (http://plntfdb.bio.uni-potsdam.de) was used for identification and classification of transcriptional factors. All the unigenes were searched against Nr, Nt, Swiss-Prot, KO and KOG databases using the BLAST algorithm (*E*-value <1E-5). On the basis of Nr annotation, the Blast2GO program were used to perform GO functional classification [98]. When a unigene could not found in any of the above databases, ESTScan was used to decipher its coding regions and sequence orientation orders [99]. GO enrichment analysis was identified with the BiNGO plugin for Cytoscape, using a hypergeometric test after Benjamini and Hochberg FDR correction (*p* <0.01) [100]. KEGG pathway enrichment analysis was done using KOBAS software (KOBAS, Surrey, UK) [101].

### Small RNAs sequencing and analysis

For sRNA library construction, 3 μg of total RNA per sample was used for the RNA sample preparations. Sequencing libraries were generated using NEBNext Mulriplex Small RNA library Prep Set for Illumina (NEB, USA). After cluster generation, the library preparations were sequenced on an Illumina Hiseq 2000 platform and 50bp single-end reads were generated. After filtering out the impure sequences (adaptor sequences and the low quality reads) through custom Perl scripts, the cellular structural RNAs, such as tRNAs, rRNAs and snoRNAs, were removed using in-house Python scripts. The clean reads were mapped to reference sequence by Bowtie [102] without mismatch to analyse their expression and distribution on the reference transcriptome.

### Identification of conserved and novel miRNAs

Mapped small RNA tags were then compared with plant mature miRNA sequences downloaded from miRBase (Release 20.0, http://www.mirbase.org/) [30] to identify conserved miRNAs. The available software miREvo [103] and mirdeep2 [104] were integrated to predict novel miRNA through exploring the secondary structure, the Dicer cleavage site and the minimum free energy of the small RNA tags unannotated in the former steps. Since there is no tea plant miRNA data set in the miRBase, we provided additional serial names for miRNAs whose number has been taken by other species in the miRBase (S5 Table).

### miRNA target prediction

Conserved and novel miRNAs, and transcriptomic unigenes datasets were used for miRNA target genes prediction by psRobot_tar scripts in psRobot [105].

### Differential expression of miRNA

Differential expression analysis of two samples was performed using the DEGseq (2010) R package. *P*-value was adjusted using *q*-value [106]. *Q*-value <0.01 and log2-fold change >1 was set as the threshold for significantly differential expression by default.

### Correlation analysis

In order to define all the possible miRNA-mRNA interactions, including positive and negative relationships between miRNA and mRNA expression, we use an in-house R script to construct miRNA-mRNA regulatory network. Briefly, normalized all the sample-matched miRNA and mRNA sequencing data; then integration of DE miRNAs with DE mRNAs was achieved by integrating expression profiles of miRNA and mRNA, sample categories and miRNA-targeting information to control for false discovery rates.

### qRT-PCR validation for DE miRNAs and mRNAs

The expression profiles of 6 *C*. *sinensis* mature miRNAs among the miRNA-mRNA interaction network were further validated using qRT-PCR. The sRNAs were isolated using a miRNA Purification Kit (CW0627, Beijing CoWin Bioscience). Synthesis of the first strand cDNA was performed with SuperRT cDNA Kit (CW0741, Beijing CoWin Bioscience) using a 2 μl of miRNA sequence-specific stem-looped RT-PCR primers, and 5.8S rRNA served as an internal control. qRT-PCR was performed in triplicate on the diluted cDNA combined with the miRNA Real-Time PCR Assay Kit (CW2142, Beijing CoWin Bioscience) with the addition of 1 μl of the appropriate forward and reverse primers. Samples were amplified using the LightCycler 480 Real-Time PCR System (Roche Applied Science) under the following parameters: 95°C for 10 min, 40 cycles at 95°C for 15 s, 60°C for 1 min. To verify RNA-seq results, 12 unigenes were selected for qRT-PCR test. qRT-PCR was performed using SYBR Premix Ex Taq II kit (Takara) and run on LightCycler 480 Real-Time PCR System (Roche Applied Science) under the following parameters: 95°C for 30 s, 40 cycles at 95°C for 5 s, 60°C for 30 s. Triplicates of each reaction were performed, and GAPDH sequence was used as endogenous control. CT values obtained through qRT-PCR were analyzed using 2^−ΔΔCT^ method to calculate relative fold change values [107]. The details of the primers used in qRT-PCR are given in S9 Table.

### Availability of supporting data

The data set supporting the results of this article is available in the NCBI SRA (Sequence read archive, http://www.ncbi.nlm.nih.gov/sra/) repository under the accession number of SRP051838.

## Supporting Information

S1 Fig Length distribution of transcript and unigene. (TIF)

S2 Fig Functional annotation and classfication of *C*. *sinensis* transcriptome. (TIF)

S3 Fig miRNA nucleotide bias at each position. (TIF)

S1 Table List of differentially expressed mRNAs of *C*. *sinensis* in CT and FT samples. (XLS)

S2 Table Enriched GO terms of differentially expressed mRNAs in CT and FT samples. (XLS)

S3 Table Pathways identified in CT and FT samples. (XLS)

S4 Table Statistics of small RNA sequences from the individual libraries. (XLS)

S5 Table List of know-miRNA and novel-miRNA of *C*. *sinensis*. (XLS)

S6 Table List of differentially expressed miRNA of *C*. *sinensis* in CT and FT samples. (XLS)

S7 Table List of miRNAs and their predicted target genes. (XLS)

S8 Table List of miRNA-mRNA pairs. (XLS)

S9 Table Sequences of primers used for the reverse transcription and quantitative real-time PCR experiments. (XLS)
