# Supplementary figures and images for: Integrated RNA-Seq and sRNA-Seq Analysis Identifies Chilling and Freezing Responsive Key Molecular Players and Pathways in Tea Plant (Camellia sinensis)
Source: PLoS One. 2015 Apr 22;10(4):e0125031. doi: 10.1371/journal.pone.0125031 (PMC4406609; doi:10.1371/journal.pone.0125031)

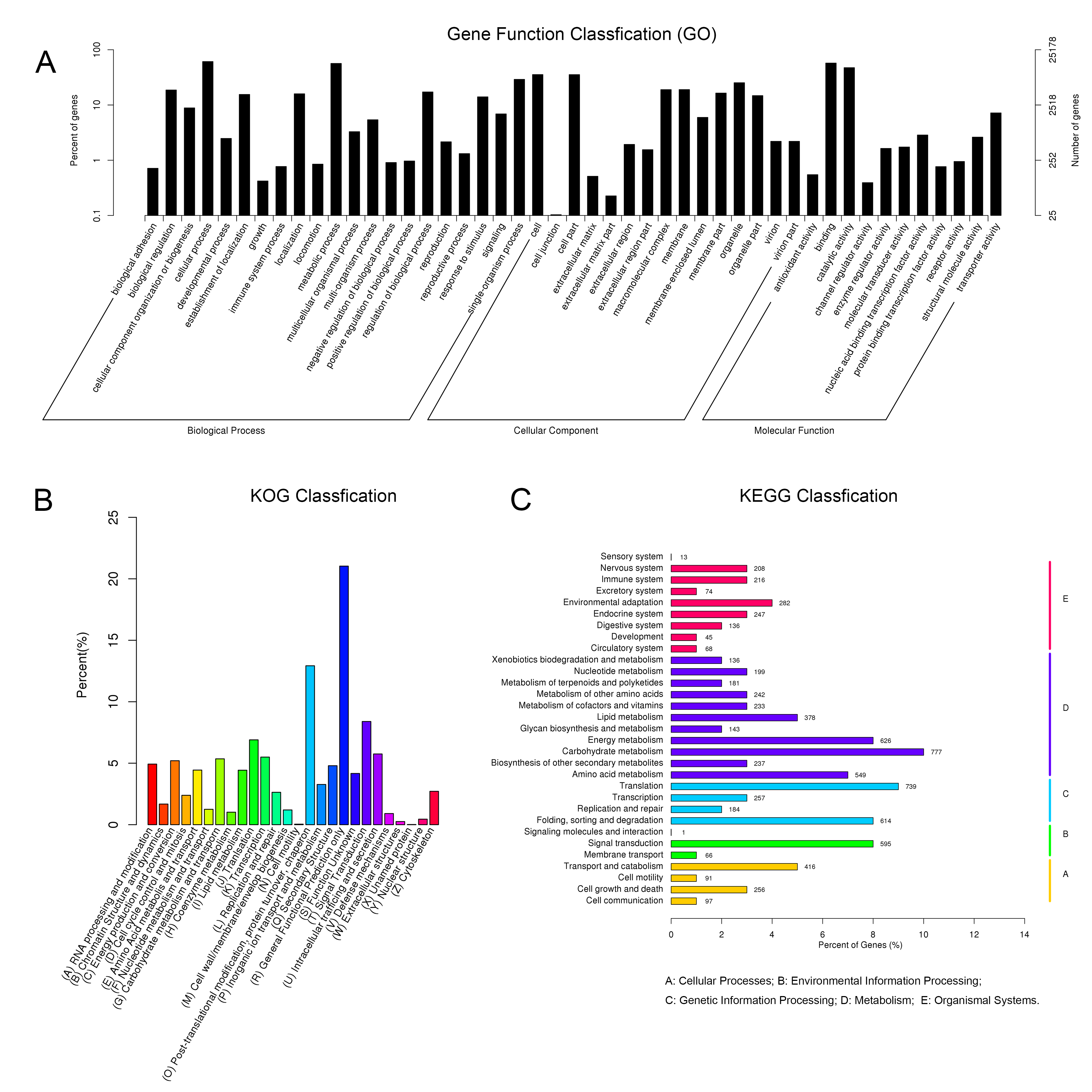

Supplement: S1 Fig — (TIF) [file pone.0125031.s001.tif]

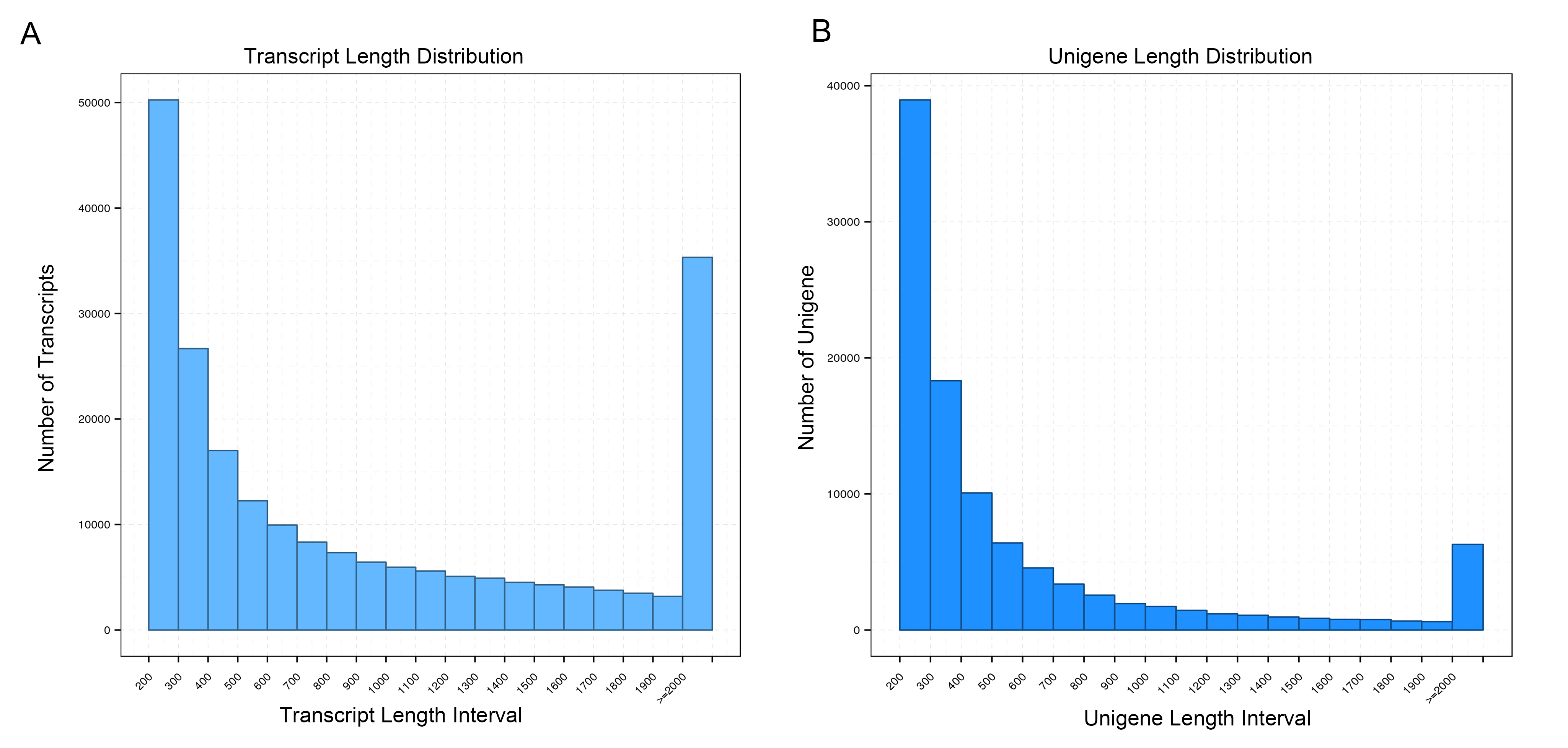

Supplement: S2 Fig — (TIF) [file pone.0125031.s002.tif]

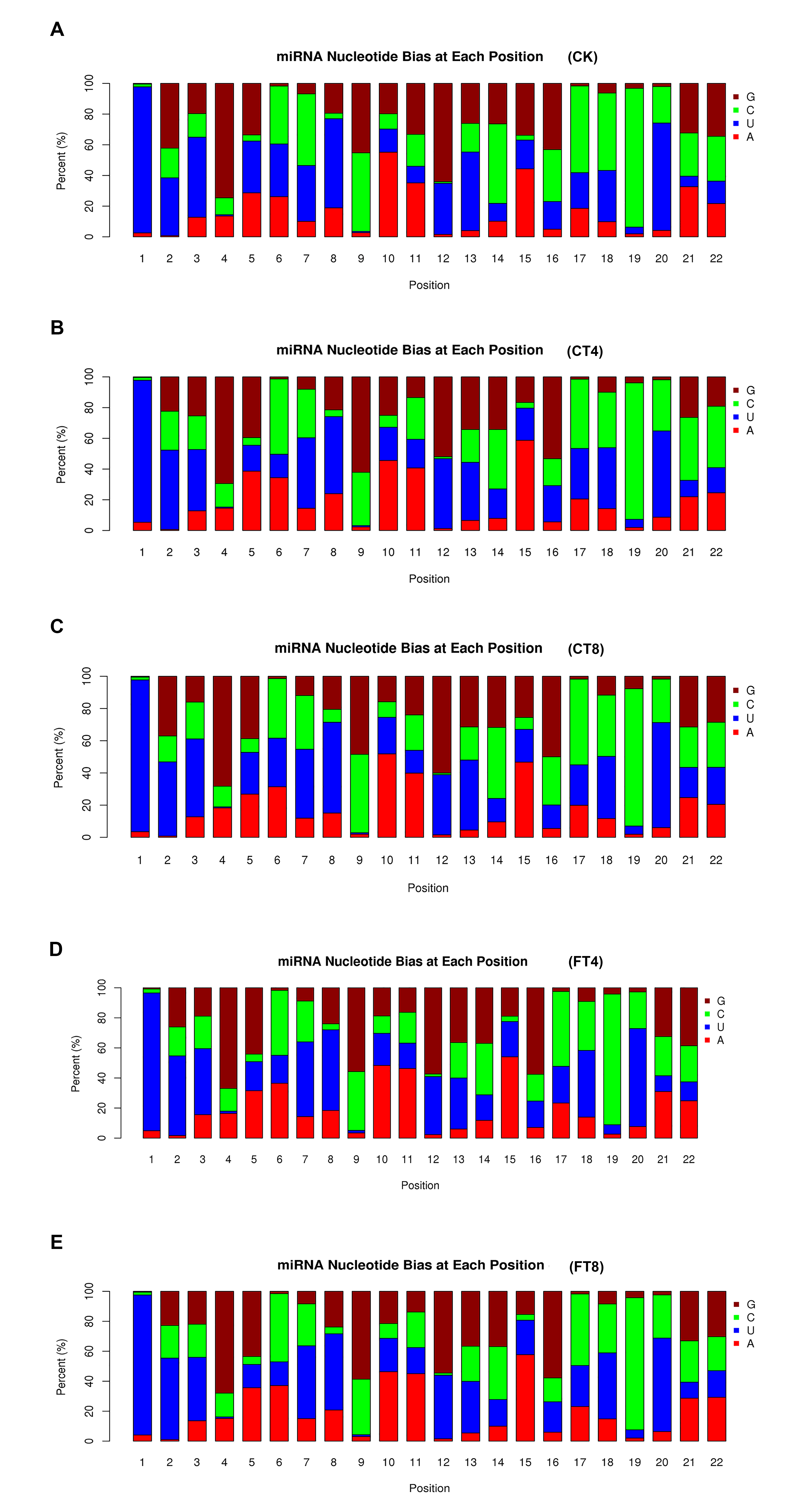

Supplement: S3 Fig — (TIF) [file pone.0125031.s003.tif]
